# Skyline Query Processing in Sensor Network Based on Data Centric Storage

**DOI:** 10.3390/s111110283

**Published:** 2011-10-28

**Authors:** Seokil Song, Yunsik Kwak, Seokhee Lee

**Affiliations:** 1 Department of Computer Engineering, Chungju National University, 72 Daehak-ro, Chungju-si, Chungbuk 380-702, Korea; E-Mail: yskwak@cjnu.ac.kr; 2 School of Media Engineering, Dong-Ah Institute of Media and Arts, Dong-Ah yedae Rd. 47 Samjuk-myeon, Anseong-si, Gyeonggi-do 456-717, Korea; E-Mail: seoklee@dima.ac.kr

**Keywords:** skyline query, sensor network, data centric storage

## Abstract

Data centric storages for sensor networks have been proposed to efficiently process multi-dimensional range queries as well as exact matches. Usually, a sensor network does not process only one type of the query, but processes various types of queries such as range queries, exact matches and skyline queries. Therefore, a sensor network based on a data centric storage for range queries and exact matches should process skyline queries efficiently. However, existing algorithms for skyline queries have not considered the features of data centric storages. Some of the data centric storages store similar data in sensor nodes that are placed on geographically similar locations. Consequently, all data are ordered in a sensor network. In this paper, we propose a new skyline query processing algorithm that exploits the above features of data centric storages.

## 1. Introduction

Recent advances in wireless communication technology, computing equipment manufacturing technology and sensor technology have led to the development of low cost, low power, multifunctional sensor nodes. Sensor nodes are able to gather physical environmental data and also to process data. Sensor nodes equipped with various sensors, such as temperature, humidity, acceleration and gases, allow monitoring of different environments. They are also able to network with other sensor nodes and exchange data with each other and external users. Sensor networks can be used in various applications, including wireless data acquisition, machine monitoring and maintenance, smart buildings and highways, environmental monitoring, site security, automated on-site tracking of expensive materials, safety management, and in many other areas [1,2].

Applications based on sensor network require various types of query processing. For example, we can deploy a sensor network to monitor air pollution of a certain city. The sensor network consists of sensor nodes that sense poisonous gases like CO_2_ and SO_2_. Users may search the geographic area in which are within a certain range or specific value. This type of query can be divided into range queries or exact match queries. Users may also need to find out the most or least polluted area in terms of CO_2_ and SO_2_. The places with high concentrations of either CO_2_ or SO_2_ are regarded to suffer serious air pollution. This type of query is called a skyline query. A skyline query on the sensor network can identify such places for environmental monitoring purposes. Skyline queries are popular in modern databases for multi-criteria decision making that has been received much attention recently among the database community.

For a past decade, various research topics on data query processing techniques in sensor networks have been proposed [3–11]. The proposed method takes carefully into account the resource constraints of sensor nodes when designing data processing algorithms. Especially, they consider the low battery power constraint as the most important factor because when some sensor nodes in a sensor network run out of energy, the sensor network may not work anymore. Accordingly, researchers have put great effort on increasing the sensor network lifetime of their data processing algorithms

Even though a sensor network is required to process various types of queries, to our knowledge existing methods only focus on one type of query. Data centric storage (DCS) is a well-known data storage technique in sensor networks that efficiently supports multi-dimensional range queries and multi-dimensional exact match queries. DCS stores data in a sensor network by its values. Each sensor reading (event) is mapped to an owner sensor node by a hashing function based on the values of the event’s attributes. The event is routed to the owner node from the original sensor node according to some routing protocols, such as greedy perimeter stateless routing (GPSR) [12]. Therefore, all events with the same value are stored at the same owner node.

**Figure 1.**
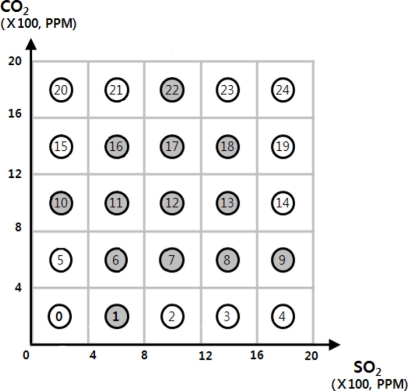
Example of GDCS.

In some of existing DCSs such as DIM [3], KDDCS [4] and GDCS [7], similar data is stored in geographically adjacent sensor nodes. On these DCSs, data are ordered by their values. This property enables DCSs to process multidimensional range queries efficiently. For example, in Figure 1, there are 25 sensor nodes that sense CO_2_ and SO_2_. In this example, each sensor node has its geographical location, and each sensor reading is mapped to geographical location by its value. The sensor reading is transmitted to a sensor node that is nearest from the mapped location. In the figure, sensor node 1 stores CO_2_ values within 0 and 4, and SO_2_ values within 4 and 8. The gray circles store some sensing values while white circles do not have any sensing values. As mentioned above, this kind of DCS are built to process multidimensional range queries efficiently. However, it is also useful to process skyline queries. In Figure 1, the most polluted area can be found by read the values stored in sensor nodes 22, 18, 13 and 9 since these sensor nodes represent the most polluted area’s sensor readings.

In this paper, we propose a skyline query processing method based on DCSs. The proposed skyline query processing method exploits the characteristic of DCSs that sensor readings are geometrically ordered on a sensor network. Consequently, the proposed method reduces the number of message transmissions for skyline query processing. In addition it also allows multidimensional range queries or exact match queries to be processed simultaneously without any change.

This paper is organized as follows. In Section 2, existing DCS methods in sensor networks are described. Also, in this section, we explain the existing skyline queries. In Section 3, the proposed skyline query processing method based on DCSs is described in detail. In Section 4, the proposed method is evaluated through simulation, and Section 5 gives the conclusions of this paper.

## 2. Related Work

In-network aggregation query processing methods used in sensor networks such as TAG [13] only send the aggregated results inside the sensor network so as to reduce the number of messages. It enables one to increase sensor network lifetime by reducing energy consumption. Some special aggregation queries such as SUM, MIN and MAX, are more effective in saving energy since they only aggregate a single aggregation value instead of all possible data. Also, DCSs process queries in a sensor network, and only send query results to a server. However, skyline queries exclude values only when data are dominated by other data, so it is difficult to find complete query results without inspecting all the data. Therefore, to process skyline queries in sensor network, it is important to establish criteria to exclude unnecessary data from the results.

Several skyline query processing methods such as [8–11] have been proposed. Most of them focus on designing filters to exclude as much unnecessary data as possible. Huang *et al.* [8] dealt with a constrained skyline query problem on MANETs by devising a single point filter-based evaluation algorithm that is easily extended to sensor networks. Xin *et al.* [10] proposed two filter-based algorithms. One is the single point filter-based algorithm TF and another is the grid filter-based algorithm GI. The TF algorithm chooses the point that dominates the maximum number of points as the filter, assuming that the data distribution density is given beforehand, while the GI algorithm exploits the grid partition of data space and generates a grid filter.

Liang *et al.* [9] proposed a new filter-based algorithm which consists of multiple rather than single points as the filter, whereby each sensor sends part of its local skyline points chosen by a greedy algorithm to its parent and the root broadcasts the received points as the global certificate obtained through in-network aggregation. The points that cannot pass through the certificate will be filtered out from transmission. Xin *et al.* [10] proposed a density function based skyline query processing algorithm. It assumes that the density function of data is known beforehand. However, in real applications it is hard to find out the density function beforehand. Chen *et al.* [11] proposed two algorithms for evaluating skyline queries are devised, which find the skyline points progressively. It partitions the dataset into disjoint subsets, followed by returning the skyline points through examining each subsequent subset progressively, using some found skyline points so far as a filter to filter out those unlikely skyline points in the currently processing subset from transmission.

## 3. Proposed Skyline Query Processing Method Based on DCS

### 3.1. Problem Definition

In this paper, we propose a skyline query processing method based on DCSs which geographically order sensor readings across a sensor network. As shown in Figure 1, finding skyline query results in such DCSs looks like a straightforward problem. If all sensor nodes store sensor readings, we only need to scan boundary sensor nodes such as sensor nodes 4, 9, 14, 19, 20, 21, 22, 23 and 24 to find the most polluted area. In DCS, some sensor nodes may or may not have stored data in the sensor network. Therefore, we need some additional data structures for each sensor node and a node traverse algorithm to search for correct query results. It is also important to reduce the number of messages for query processing. When broadcasting query processing messages to neighbor nodes, some unnecessary messages can be transmitted, and a sensor node may receive same messages repeatedly. We carefully take into account the mentioned problems in our proposed skyline query processing method. Our proposed query processing method will be described in detail in the next subsection.

### 3.2. Proposed Skyline Query Processing Method

The proposed method is also based on the following assumptions: first, a DCS is built on a sensor network and sensor readings are geographically ordered across the sensor network like KDDCS, GDCS and DIM. Second, all the sensor nodes in a sensor network have a unique identifier (ID). Third, they know their geographic locations and their neighbor nodes’ IDs. A neighbor node refers to a node to which a sensor node can communicate within one hop. The last assumption is that each node knows the ranges of data stored in its neighbor nodes. We call the range of data stored in a neighbor node NDR. On the basis of these assumptions, a skyline query is processed in three stages.

**Figure 2.**
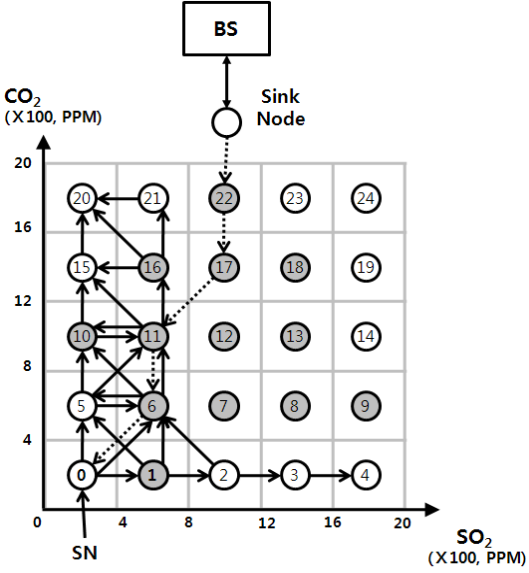
Sensor network where GDCS is deployed

To describe the proposed skyline query processing method more clearly, we use an example sensor network as shown in Figure 2. We assume that GDCS which uses virtual grid technique is built on the sensor network. In the figure, the GDCS is based on 5 × 5 grid. The sensor network is deployed to monitor CO_2_ and SO_2_. In this figure, the sensor nodes at the left-lower corner stores sensor readings whose values are smallest, while the sensor nodes at the right-upper corner store the data whose CO_2_ and SO_2_ values are largest.

In the first stage, whenever a skyline query is issued, a start node (SN) where query processing is started is selected, and then the skyline query is transmitted to the SN. For example, in Figure 2, let us assume that a skyline query “find the most polluted area” is issued. In this case, candidate query results may be stored in the sensor node 24 at the upper-right corner and its neighbor nodes. Therefore, the sensor node 24 is selected as a SN. On the other hand, if the query is “find the area in which atmospheric pollution is the least”, the sensor node 0 at the lower-left corner and its neighbor nodes may store candidate query results. For this query, the sensor node 0 is selected as a SN.

A skyline query may be given by a user, and the query is injected into the sensor network by a base station (BS). The BS analyzes the skyline query to select a SN which is expected to process the result most effectively, and transmits it to the SN. The SN really starts to process the skyline query.

The second stage is started by the SN. The SN creates a skyline query message (SQM) and sends it to propagate the SQM to neighbor nodes to search for candidate nodes (CNs) where candidate data for the query are stored. The SQM consists of (Q, CSR, SIDs, TIDs), where Q is the contents of the skyline query, and CSR is the range of candidate data set which is currently gathered, SIDs are the set of all sensor nodes’ IDs that the SQM is passed by, and TIDs are the sensor nodes’ IDs that will receive the SQM. The CSR, SIDs and TIDs of the SQM which is created by the SN may have NULL values.

The SQM transmission process is repeated until complete candidate results are found through the following process. When a sensor node (or a SN) receives a SQM, it reads the CSR of the SQM, and updates the CSR to reflect the range of data stored in the sensor node. Of course, if the sensor node does not have any data, there would be no need to change the CSR. After updating the CSR, the sensor node checks whether the CSR is NULL. If the CSR is NULL, the sensor node transmits the SQM to all the neighbor nodes since the sensor node does not dominate any other node. If the CSR is not NULL, the sensor node selects neighbor nodes which have NDRs that are not dominated by the CSR and other NDRs. The current sensor node excludes sensor nodes that transmit the SQM to it. The selected nodes are included in the TIDs of the SQM. After that, the sensor node transmits to selected sensor nodes the SQM, and the sensor nodes that receive the SQM repeat the above process.

The sensor node that transmits the SQM stores the CSR of the SQM to avoid unnecessary message transmission. As shown in Figure 2, a sensor node may receive several SQMs with the same CSR. If the sensor node processes all the SQMs with the same CSR, unnecessary message transmissions will occur. To avoid this case, each sensor node stores CSRs of SQMs that it processes. Whenever a sensor node receives a SQM, it compares the CSR of the SQM with the stored CSRs, and if the CSR of the SQM is contained in one of the stored CSRs, it ignores this newly received SQM. If not, the above process will be repeated and the sensor node stores the CSR of SQM. This message transmission process is stopped when a sensor node that receives a SQM cannot find any neighbor node that is not dominated.

The last stage is that BS gathers candidate data set from candidate sensor nodes (CNs), and generates a final query result. A CN refers that a sensor node who receives a SQM and has stored data. The CN transmits its stored data to the BS. The BS analyzes the candidate data set from CNs, and produces the final query result from the candidate set.

We describe the proposed skyline query process with an example in Figure 2. In Figure 2, gray circles represent sensor nodes that have stored sensor readings, and white circles are sensor nodes without stored data. The BS receives a skyline query “find out the area in which pollution degree is the least”, and analyzes the skyline query to find out a SN. In this example, sensor node 0 at the left-lower corner is selected, and the BS sends the skyline query to sensor node 0.

Sensor node 0 creates a SQM (Q, NULL, NULL, NULL), and checks if itself stores data. Since sensor node 0 does not store data, the CSR of the SQM remains as NULL. Therefore, it sends the SQM (Q, NULL, 0, {1, 5, 6}) to all neighbor nodes 1, 5 and 6. Sensor node 1 receives the SQM and changes the CSR of the SQM to {(4, 0), (8, 4)} because it stores data within the range from (4, 0) to (8, 4). Then, the sensor node compares the changed CSR with NDRs of its neighbor nodes 2, 7, 6, 5, and 0. In that comparison, the sensor node checks that the data range of the CSR is dominated by the NDRs of the neighbor nodes. Through the comparison, sensor nodes 0, 2, 5 and 6 are selected, but the 0 is excluded because 0 is included in the TIDs of the SQM. Sensor node 1 transmits a SQM (Q, {(4, 0), (8, 4)}, {0, 1}, {2, 5, 6}) to sensor nodes 2, 5 and 6.

Sensor node 6 receives the SQM from sensor node 0, and it updates the CSR of the SQM to {(4, 4), (8, 8)} to include its stored data range. After that, it compares the CSR with the NDRs of its neighbor nodes, 0, 1, 2, 5, 7, 10, 11 and 12 to find out NDRs that are not dominated by the CSR. Since node 12 is explicitly dominated by the CSR, it is excluded. Consequently, sensor nodes 0, 1, 2, 5, 10, 11 and 7 are remained, but the NDR of sensor node 7 is dominated by the NDR of sensor node 1, sensor node 7 is excluded. Finally, sensor node 6 transmits a SQM (Q, {(4, 4), (8, 8)}, {0, 6}, {0, 1, 2, 5, 10, 11}) to sensor nodes 0, 1, 2, 5, 10 and 11, and it stores the CSR {(4, 4), (8, 8)}.

Simultaneously, sensor node 6 receives the SQM from sensor node 1, and it changes the CSR {(4, 0), (8, 4)} of the SQM to include the stored data range to {(4, 0), (8, 8)}. Then, through comparing the updated CSR with NDRs of neighbor nodes 0, 1, 2, 5, 7, 10, 11 and 12, it selects sensor nodes which have NDRs that are not completely dominated. Consequently, sensor nodes 0, 1, 5, 10 and 11 are selected, but sensor nodes 0 and 1 are excluded since it is included in the SIDs of the SQM. Finally, sensor nodes 5, 10 and 11 are selected. Then, sensor node 6 compares the locally stored CSR with the CSR of the SQM. In this case, the local CSR is not included in the SQM’s CSR, so the SQM (Q, {(4, 0), (8, 8)}, {0, 1, 6}, {5, 10, 11}) is transmits to selected neighbors.

Sensor node 11 receives the SQM (Q, {(4, 0), (8, 8)}, {0, 1, 6}, {5, 10, 11}) from sensor node 6, and after the same process, sends the updated SQM to 10, 15 and 16. It stores the CSR {(4, 0), (8, 12)} of the SQM locally. Then, sensor node 11 receives the SQM (Q, {(4, 0), (8, 8), (0, 8), (4, 12)}, {0, 1, 6, 10}, {11, 15}), and update the CSR of the SQM to {(4, 0), (8, 12), (0, 8), (4, 12)}. It compares the local CSR with the updated CSR. In this case, the updated CSR is included in the local CSR, so the sensor node does not transmit any SQM to its neighbors.

Each sensor node that receives SQMs repeats the above process and sensor nodes 1, 6, 10, 11 and 16 becomes CNs. The CNs transmit their stored data to the BS immediately after processing SQM, and then the BS generates a final query result set from the received candidate data.

## 4. Performance Evaluation

We show the performance of the proposed skyline query processing method through simulation. Some skyline query processing methods in sensor networks have been proposed, but none of them are based on DCSs. Consequently, instead of comparing our method with existing skyline query processing methods, we measure how much energy is consumed additionally after a DCS is built on a sensor network.

In this simulation, we use GDCS as a DCS, and the simulation parameters are shown in Table 1. We assume that the sensor network is deployed in a 200 × 200 m^2^ region. Within the region, there are 400 randomly located sensors. The initial energy level of each sensor node is 100, and the energy consumption level for a message transmission is 0.1, and receiving a message consumes 0.05 units of energy level. All sensor nodes have the same transmission range (15 m).

All nodes collect 100 sensor readings of normal distribution, and store them inside sensor network by using GDCS. The 80% of the events falls into the 20% of the reading range, and in 1%–25% of nodes (20 sensor nodes) do not store sensor reading. We generate 50 skyline queries and send the queries to SNs in the sensor network. We exclude the communication cost from the BS to each SN. Consumed energy level is calculated with measured communication cost for SQM transmissions between sensor nodes and candidate data transmissions from CNs to the BS.

Simulation results are shown in Table 2 when the number of sensor nodes that do not store sensor readings is 20. The table shows the number of sensor nodes that participate in query processing, the average energy level of a sensor network consumed for skyline query processing, the average energy level of a sensor node consumed for processing a skyline query, the average energy level of a sensor node to maintain GDCS and the average energy level of a sensor node to maintain GDCS and process a skyline query. As shown in Table 2, the nodes which participated in query processing are only 55, or just 14% of all sensor nodes. Also, the average energy level consumed additionally to process a skyline query is only about 0.5 units.

**Figure 3.**
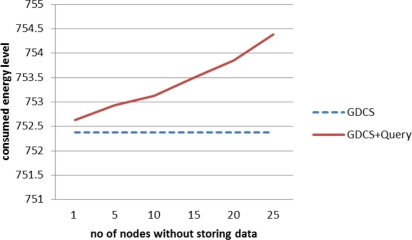
Energy consumption with varying the number of node without storing data.

The proposed algorithm is affected by the number of sensor nodes without storing data. Figure 3 shows the energy level consumed to process skyline query when the number of sensor nodes not storing sensor readings varies. As the number of sensor nodes without data increases, the energy consumption increases. If a SN and the sensor nodes near the SN do not store data, the number of SQM transmissions increases.

## 4. Conclusions

In this paper, we propose an energy efficient skyline query processing method based on DCSs in which data are geographically ordered across a sensor network. The proposed skyline query processing method exploits the property of DCSs to reduce the number of message transmissions. Through simulation, only 13.75% of sensor nodes participated in query processing and just 4 units of energy are consumed to process a skyline query. However, when the number of sensor nodes without data increases, the energy consumption for query processing increases. In our future work, we will study how to apply the proposed method continuous skyline query processing.

## Figures and Tables

**Table 1.** Simulation parameters.

Parameters	Values

Number of sensor nodes	400
Region of sensor network	200 × 200 m^2^
Initial energy of node	1,000 units
Energy consumption required for a message transmission	0.1 units
Energy consumption required for receiving a message	0.05 units
Storage capacity	100 units
Transmission range of a sensor node	15 m

**Table 2.** Energy consumption while query processing.

Energy level	Measured value

Number of sensor nodes participated in skyline query processing	55 numbers of nodes (13.75%)
Total energy level consumed for skyline query processing	220.55 units
Average energy level of a participating sensor node consumed for skyline query processing	4.01 units
Average energy level of a sensor node to maintain GDCS	752.38 units
Average energy level of a sensor node (GDCS maintenance + skyline query processing)	752.93 units
